# Association between gut microbiota and influenza: a bidirectional two-sample mendelian randomization study

**DOI:** 10.1186/s12879-023-08706-x

**Published:** 2023-10-17

**Authors:** Fan Xu, Xiuyuan Gan, Yang Tao, Dongling Li, Puguang Xie, Fangying Liu, Fan Yang, Yu Ma

**Affiliations:** 1 Chongqing Key Laboratory of Emergency Medicine, Chongqing Emergency Medical Center, School of Medicine, Chongqing University Central Hospital, Chongqing University, Chongqing, 400014 China; 2 Central laboratory of Chongqing Emergency Medical Center, Chongqing University Central Hospital, Chongqing, 400014 China; 3 Department of Critical Care Medicine, Chongqing University Central Hospital, Chongqing, 400014 China

**Keywords:** Two-sample mendelian randomization, Influenza, Pneumonia, GWAS

## Abstract

**Background:**

Previous observational studies have indicated a correlation between the gut microbiota and influenza; however, the exact nature of the bidirectional causal connection remains uncertain.

**Method:**

A two-way, two-sample Mendelian randomization (MR) study was conducted to evaluate the possible causal connection between the gut microbiota and the two outcomes of influenza (pneumonia without influenza and influenza pneumonia). The statistical analysis of gut microbiota is derived from the information of the most extensive meta-analysis (GWAS) conducted by the MiBioGen Alliance, encompassing a sample size of 18,340.The summary statistical data for influenza (not pneumonia, n = 291,090) and influenza pneumonia (n = 342,499) are from GWAS data published by FinnGen consortium R8.Estimate and summarize Single-nucleotide polymorphisms (SNPs) using Inverse variance weighted (IVW), MR Egger, and Weighted median (WM) in bidirectional MR analysis. To assess the heterogeneity, horizontal pleiotropy, and stability of SNPs, we employed Cochran’s Q test, MR Egger intercept test, and sensitivity analysis.

**Result:**

The IVW analysis indicated that there was a significant association between influenza infection and five bacterial taxa. Additionally, the abundance changes of seven gut microbiota were found to be causally related to influenza infection. In addition, seven bacterial taxa showed a significant association with the occurrence of influenza pneumonia. The findings from the WM analysis largely support the outcomes of IVW, however, the results of MR egger analysis do not align with IVW. Furthermore, there is no proof to substantiate the cause-and-effect relationship between influenza pneumonia and the composition of gut microbiota.

**Conclusion:**

This analysis demonstrates a possible bidirectional causal connection between the prevalence of particular gut microbiota and the occurrence of influenza infection. The presence of certain gut microbiota may potentially contribute to the development of pneumonia caused by influenza. Additional investigation into the interaction between particular bacterial communities and influenza can enhance efforts in preventing, monitoring, and treating influenza.

**Supplementary Information:**

The online version contains supplementary material available at 10.1186/s12879-023-08706-x.

## Introduction

Influenza is a respiratory infectious disease caused by influenza viruses [1]. Epidemiology has shown a correlation between influenza and pneumonia, where the peak time of pneumonia closely aligns with the peak time of influenza [2]. Influenza-induced pneumonia can manifest as primary viral pneumonia or secondary bacterial pneumonia [1]. Hospitalized patients with influenza-induced pneumonia have higher mortality rates, contributing significantly to the global burden of pneumonia [3].

Despite the assistance of vaccines and antiviral medications in lessening the consequences of influenza, the virus’s capacity to mutate and the restricted efficacy of antiviral drugs continue to present difficulties [4]. The emergence of new subtypes, such as the H5 subtype derived from highly pathogenic avian influenza, further underscores the need for effective prevention, treatment, and monitoring strategies [5].

Scientists have found that the microbial community in the gut influences the body’s immune reaction to viral infections, such as influenza [6]. For example, by increasing the number of beneficial bacteria in the gut through supplementation, it is possible to enhance the immune system’s ability to fight against influenza viruses [7]. On the other hand, the utilization of antibiotics can disturb the equilibrium of intestinal microorganisms and diminish the safeguarding impact of influenza vaccines [8]. These findings suggest that studying and utilizing specific gut microbiota could be a promising approach for preventing and treating influenza [6–8].

Furthermore, studies have revealed that influenza virus infection can lead to distinct changes in the gut microbiota, differentiating it from bacterial infections, COVID-19 infections, and other viral infections [9, 10]. Hence, tracking alterations in the gut microbiota of individuals may function as an indicator for accurately detecting influenza and differentiating it from other diseases.

Thus, it is essential to comprehend the connection among influenza, pneumonia, and the gut microbiome in order to devise approaches that lessen the impact of influenza and enhance patient outcomes. Nevertheless, the relationship between intestinal microbiota and invasive viruses is intricate and requires additional examination. To explore the bidirectional causal relationship between the susceptibility and intensity of influenza and the gut microbiota, we utilized a two-sample Mendelian randomization (MR) analysis. Our study utilized extensive datasets with two distinct influenza outcomes: (1) influenza (excluding pneumonias), and (2) influenza-induced pneumonias. The utilization of genetic variation in MR analysis aids in the creation of exposure tools and the assessment of the causal association between exposure and outcomes [11]. The reasonable causal order is ensured as the genetic variation and outcomes association remains unaffected by other confounding factors due to the random distribution of genotypes from parents to offspring [12].

## Method

### Summary statistics from a genome-wide association study (GWAS)

Publicly accessible GWAS studies provided the acquired summary data.The summary information on gut microbiota is derived from the GWAS investigation conducted by the International MiBioGen Alliance [13], encompassing 24 cohorts and 18,340 individuals. An analysis was performed to localize quantitative microbiome trait loci (mbQTL) on each cohort, taking into account only taxonomic groups that were found in over 10% of the samples. This analysis yielded a total of 211 taxonomic groups, including 35 families, 20 orders, 16 classes, 9 phyla, and 131 genera. Furthermore, the analysis of mapping binary trait loci (mbBTL) encompasses taxonomic categories that are present in the included samples within a range of 10–90%.

The FinnGen consortium conducted a GWAS study that included data on influenza (excluding pneumonia) with 4471 cases and 286,619 controls, as well as data on influenza pneumonia with 55,880 cases and 286,619 controls [14]. The influenza (non-pneumonia) cohort is considered a regular influenza cohort, while the influenza pneumonia cohort is considered a severe influenza cohort.

Each cohort included in the GWAS study received ethical approval and agreed to participate, and summary-level data were provided for analysis. Adjustments were made for gender, age, the top 10 principal components, and genotype batches. The genomic inflation factor and Linkage Disequilibrium (LD) score regression H2 were calculated to estimate the population stratification (refer to supplementary Table S1).

### Independent variables (IVs) selection criteria

MR analysis employs genetic variations as IVs to represent specific exposures, enabling causal inference between exposures and outcomes by transforming phenotype-to-phenotype causal studies into genotype studies. The advantages of MR include: genetic variations precede disease outcomes, thereby eliminating confounding biases due to reverse causality; modern biotechnologies allow for highly accurate measurement of genetic variations, substantially reducing estimation biases associated with measurement errors. Single nucleotide polymorphisms (SNPs) are the most commonly used genetic variations in MR analysis, referring to DNA sequence diversity caused by variations at the nucleotide level (transitions and transversions, with a ratio of 2:1) on the genomic level. Generally, SNPs refer to single nucleotide variations with a minor allele frequency (MAF) greater than 1%. Based on the frequency of alleles, SNPs can further be categorized into major alleles and minor alleles. The proportion of minor alleles (or the minimum allele) in a given population is known as the “minor allele frequency (MAF),” which is commonly used as a criterion for SNP selection.

The following are the guidelines for choosing IVs [15].

1. Identification of Potential Single-nucleotide polymorphisms (SNPs): Each genus’s potential IVs were identified by selecting SNPs that met a significance threshold of P < 5e-6 for the entire site [16].
2. The LD calculation between these SNPs: To ensure the independence of selected genetic variations, the LD window is commonly set to 10,000 kb with a threshold of r2<0.01. LD refers to the nonrandom association between alleles of different loci. It is assessed using two parameters, r2 and kb. The r2 value ranges from 0 to 1, with smaller values indicating a higher degree of complete linkage equilibrium between two SNPs, implying a random distribution of these SNPs. kb represents the length of the region considered for LD, as genetic loci in close proximity on a chromosome tend to be inherited together, leading to a large r2 between closely located loci. Adequate LD window size and r2 threshold are chosen to ensure independence, considering the strong influence of LD. We excluded all other SNPs within a 10,000 kb range of a given SNP that met the LD criteria of r2 < 0.001.
3. Exclusion of SNPs with low Minor Allele Frequency: SNPs having MAF less than or equal to 0.01 were eliminated from consideration.
4. Exclusion of Palindrome SNPs: Palindrome SNPs were excluded by inferring the pre-chain allele using the Allele frequency information.

These selection criteria ensure that the chosen IVs have significant associations with the research outcome and meet certain genetic characteristics necessary for reliable analysis.

### Statistical analysis

In the MR model, the instrumental variable, which represents the genetic variation, needs to satisfy three core assumptions [12, 17]: the relevance assumption, indicating a robust and significant correlation between the genetic variation (Z) and the exposure factor (X) (γ ≠ 0); the independence assumption, stating that the genetic variation (Z) is independent of confounders (U) that affect the relationship between the exposure factor (X) and the outcome (Y) (φ1 = 0); and the exclusion restriction assumption, which asserts that the genetic variation only affects the outcome through the exposure factor and not through any other pathway (φ2 = 0).

The statistical analysis involved bidirectional MR analysis in the MiBioGen and FinnGen cohorts, utilizing three distinct MR techniques relying on different assumptions: inverse variance weighting (IVW), weighted median (WM), and MR-Egger regression [12]. The main statistical model used was the IVW technique, which provided both fixed effects and random effects IVW approaches. IVW is a method of aggregating two or more random variables to minimize the total variance. The weight assigned to each random variable in the sum is inversely proportional to its variance. The variance is often used to combine results from independent studies. We employed the Wald ratio method to calculate the exposure-outcome effect size for each SNP. At first, the fixed effects IVW method was utilized to calculate causal estimates by meta-analyzing Wald ratio estimates for each instrumental variable. When there was noticeable heterogeneity (P < 0.05), the random effects IVW approach was employed [17]. To ensure the accuracy of the results, multiple methods were employed, including MR-Egger regression, weighted median.

To ensure the fulfillment of the three critical assumptions in MR analysis, we need to conduct the following three aspects of sensitivity analysis to evaluate the robustness of the results, the reliability of the conclusions, and the presence of potential biases, such as pleiotropy (which refers to a gene influencing multiple phenotypes) and data heterogeneity. Moreover, we also assess whether a particular instrumental variable has a significant impact on the outcome variable, typically using the “leave-one-out” method.

To evaluate heterogeneity, the Cochran’s Q test was utilized, and both the fixed effects IVW approach and MR-Egger regression were applied to identify heterogeneity in causal estimates [12].The odds ratio (OR) was obtained by transforming the combined effect estimate β through the formula β = ln(OR). And 95% confidence interval (CI) of OR was caculated further. Cochran’s Q statistics were employed for quantifying heterogeneity, where a P-value below 0.05 was deemed as an indication of substantial heterogeneity.

To assess potential pleiotropic effects of instrumental variables, the MR-Egger regression method was employed. We investigated the existence of directional horizontal pleiotropy in the causal estimates by analyzing the intercept term in the MR-Egger regression [17].

Furthermore, a leave-one-out analysis was performed to detect any possible anomalous instrumental variables. This was achieved by excluding each SNP one at a time and conducting MR analysis on the remaining SNPs.

R version 4.2.2 (R Foundation for Statistical Computing, Vienna, Austria) was utilized for all statistical analyses. The TwosampleMR (version 0.5.6), data.table (version 1.14.8), tidyverse (version 1.3.2), writexl, and readxl R packages were utilized for conducting MR analyses.

## Result

### The relationship between gut microbiota and Influenza (not- pneumonia) in terms of cause and effect

**Fig. 1:**
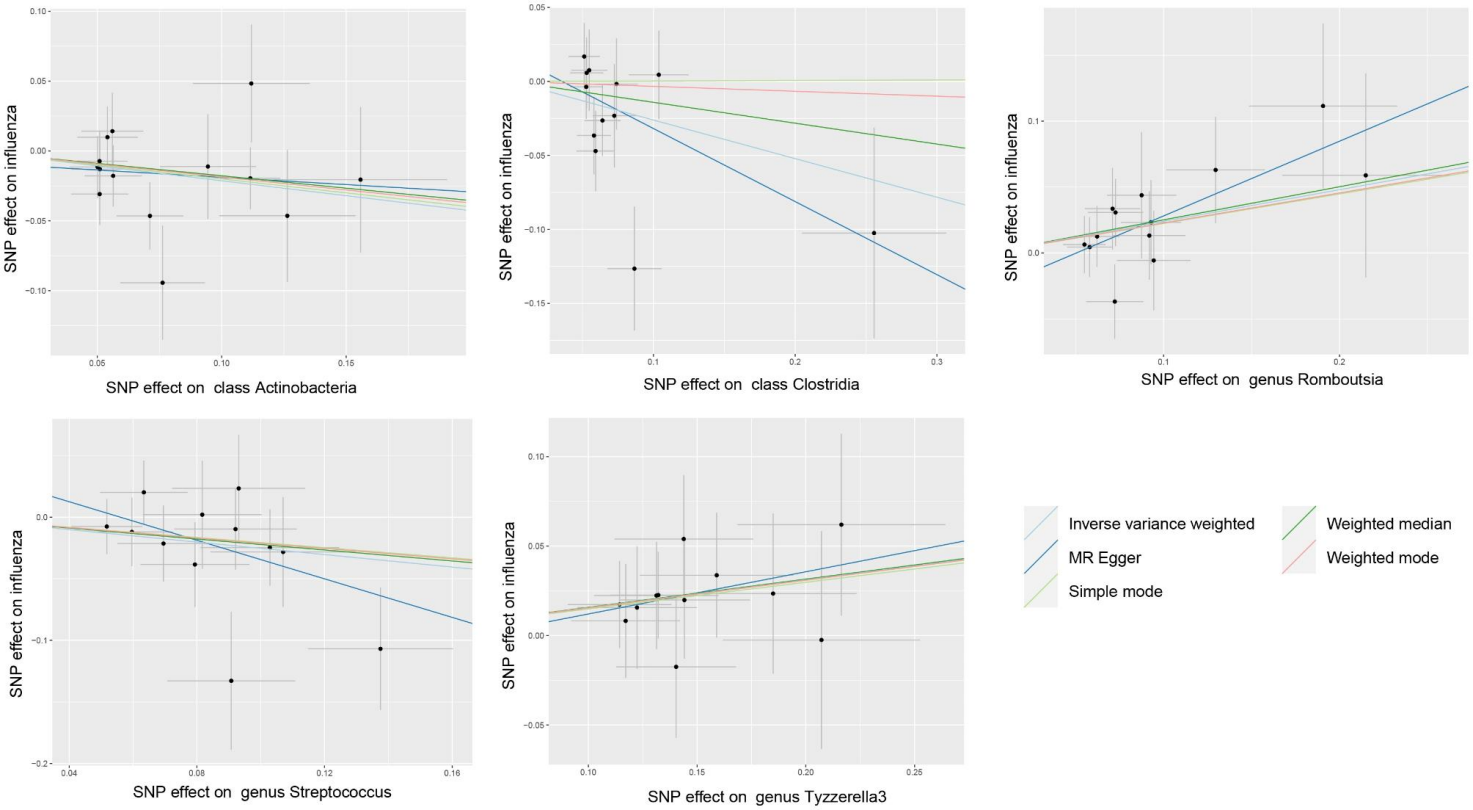
Scatter plots for the impact of gut microbiota on influenza (not-pneumonias) outcomes

Initially, our research examined the cause and effect relationship between the gut microbiome and the outcome of influenza infection. At first, employing the IVW technique, we detected five bacterial categories that exhibited a significant correlation with influenza (non-pneumonia) results (refer to Table 1; Fig. 1). We observed an inverse relationship between the class Actinobacteria (OR 0.81, 95% CI 0.67 to 0.98; p-value = 0.028), class Clostridia (OR 0.77, 95% CI 0.60 to 0.99; p-value = 0.039), and genus Streptococcus (OR 0.78, 95% CI 0.61 to 0.98; p-value = 0.035) and influenza (non-pneumonia), indicating that these three bacterial categories might potentially offer protection against influenza infection. Furthermore, it was discovered that the Romboutsia genus (OR 1.27, 95% CI 1.04 to 1.56; p-value = 0.021) and Tyzzerella3 genus (OR 1.17, 95% CI 1.02 to 1.34; p-value = 0.023) exhibited a causal connection with influenza (non-pneumonia), suggesting a heightened susceptibility to influenza infection.

The WM analysis provided support for most of these findings, although Mr. Egger’s analysis did not.The Egger intercept findings suggested the lack of directional level pleiotropy (Table 1), and the absence of heterogeneity was supported by the Cochran’s Q-test (supplementary table S2). Moreover, the leave-one-out analysis revealed that none of the individual SNPs had a substantial influence on the overall causal effect (supplementary figure S1).

**Table 1:** The impact of gut microbiota on influenza (not-pneumonias) outcomes

Exposure	Method	N_IV	b	p	OR	Egger_intercept	P_pleiotropy
class Actinobacteria	IVW	14	-0.213	0.028	0.81	-0.008	0.678
class Actinobacteria	MR Egger	14	-0.104	0.711	0.90	-0.008	0.678
class Actinobacteria	WM	14	-0.178	0.189	0.84	-0.008	0.678
class Clostridia	IVW	12	-0.260	0.039	0.77	0.018	0.487
class Clostridia	MR Egger	12	-0.494	0.186	0.61	0.018	0.487
class Clostridia	WM	12	-0.141	0.376	0.87	0.018	0.487
genus Romboutsia	IVW	13	0.240	0.021	1.27	-0.029	0.270
genus Romboutsia	MR Egger	13	0.567	0.085	1.76	-0.029	0.270
genus Romboutsia	WM	13	0.251	0.075	1.29	-0.029	0.270
genus Streptococcus	IVW	12	-0.253	0.035	0.78	0.044	0.245
genus Streptococcus	MR Egger	12	-0.222	0.161	0.46	0.044	0.245
genus Streptococcus	WM	12	-0.253	0.035	0.80	0.044	0.245
genus Tyzzerella3	IVW	12	0.156	0.023	1.17	-0.012	0.835
genus Tyzzerella3	MR Egger	12	0.236	0.550	1.27	-0.012	0.835
genus Tyzzerella3	WM	12	0.158	0.072	1.17	-0.012	0.835

Abbreviations: b represents effect size, IVW refers to inverse variance weighted, MR denotes Mendelian randomization, WM stands for Weighted median, N_IV represents the number of instrumental variables, and OR stands for odds ratio

**Fig. 2:**
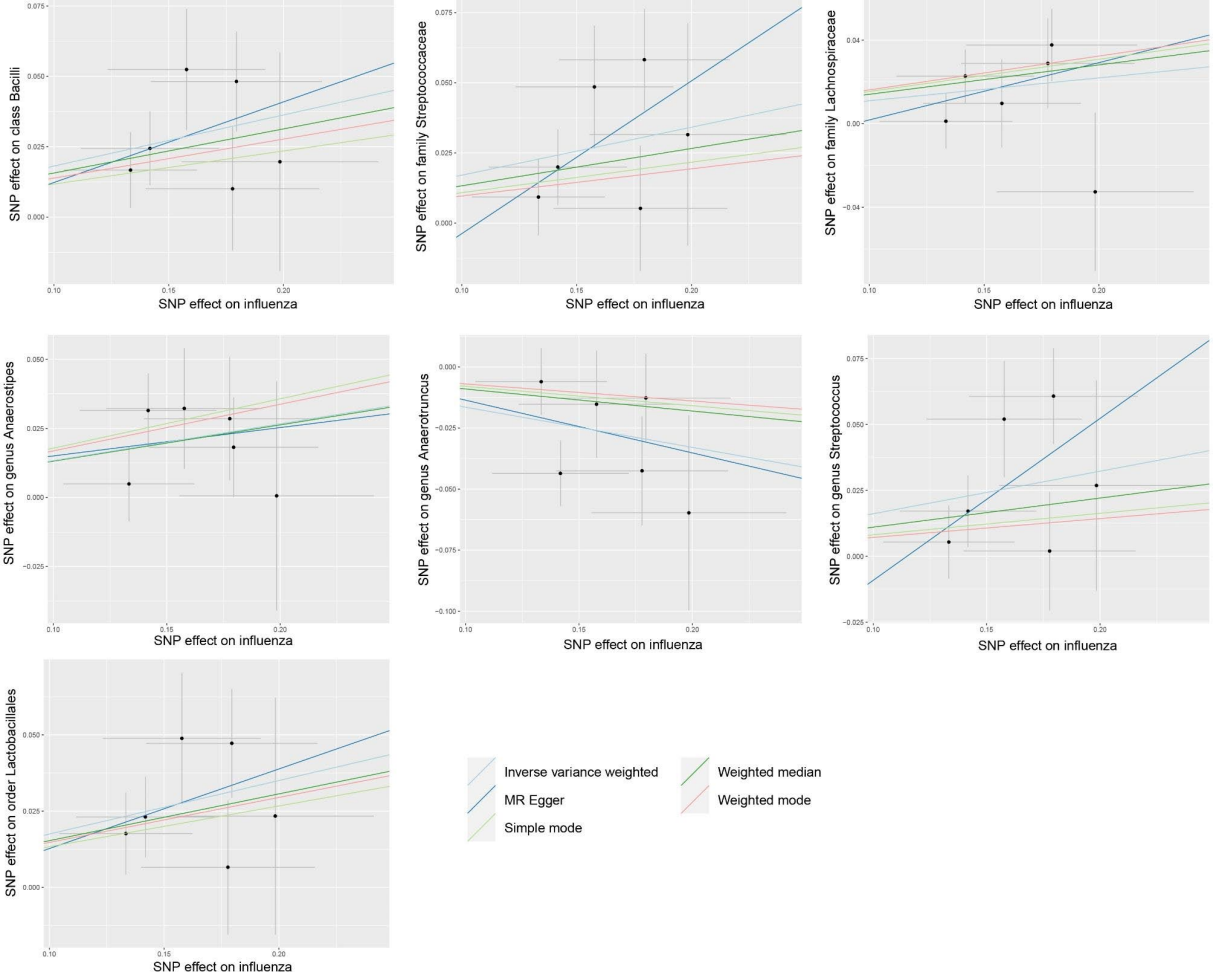
Scatter plots for the impact of influenza (not-pneumonias) on gut microbiota outcomes

In contrast, our findings from Table 2; Fig. 2 indicate that the influenza virus (non-pneumonia) causes changes in the composition of gut microbiota. A potential decrease in the abundance of the bacterial taxa Anaerotruncus was suggested by our observation of a negative correlation with influenza (not-pneumonia) (OR 0.85, 95% CI 0.77 to 0.94; p-value = 0.001). Furthermore, non-pneumonia influenza demonstrated positive connections with the Bacilli category (OR 1.20, 95% CI 1.09 to 1.31; p-value < 0.001), Lachnospiraceae family (OR 1.12, 95% CI 1.02 to 1.22; p-value = 0.016), Streptococcaceae family (OR 1.19, 95% CI 1.07 to 1.31; p-value = 0.001), Anaerostipes genus (OR 1.14, 95% CI 1.04 to 1.25; p-value = 0.005), Streptococcus genus (OR 1.18, 95% CI 1.05 to 1.32; p-value = 0.006), and Lactobacillales order (OR 1.19, 95% CI 1.09 to 1.31; p-value < 0.001). Influenza infection may lead to a decrease in the prevalence of these six taxonomic groups, according to these findings.

Again, the WM analysis supported most of these findings, but Mr. Egger’s analysis did not. The Egger intercept findings suggested the lack of directional level pleiotropy (Table 2), and the absence of heterogeneity was supported by the Cochran’s Q-test (supplementary table S3). In addition, the leave-one-out analysis indicated that none of the individual SNPs had a notable influence on the overall causal effect (supplementary figure S2).

**Table 2:** The impact of influenza (not-pneumonias) on gut microbiota outcomes

Outcome	Method	N_IV	b	p	OR	Egger_intercept	P_pleiotropy
class Bacilli	IVW	6	0.182	0.000	1.20	-0.016	0.783
class Bacilli	MR Egger	6	0.286	0.469	1.33	-0.016	0.783
class Bacilli	WM	6	0.157	0.011	1.17	-0.016	0.783
family Lachnospiraceae	IVW	6	0.110	0.016	1.12	-0.026	0.681
family Lachnospiraceae	MR Egger	6	0.274	0.506	1.32	-0.026	0.681
family Lachnospiraceae	WM	6	0.141	0.019	1.15	-0.026	0.681
family Streptococcaceae	IVW	6	0.171	0.001	1.19	-0.058	0.390
family Streptococcaceae	MR Egger	6	0.544	0.236	1.72	-0.058	0.390
family Streptococcaceae	WM	6	0.133	0.052	1.14	-0.058	0.390
genus Anaerostipes	IVW	6	0.133	0.005	1.14	0.005	0.937
genus Anaerostipes	MR Egger	6	0.103	0.794	1.11	0.005	0.937
genus Anaerostipes	WM	6	0.131	0.037	1.14	0.005	0.937
genus Anaerotruncus	IVW	6	-0.165	0.001	0.85	0.008	0.910
genus Anaerotruncus	MR Egger	6	-0.216	0.642	0.81	0.008	0.910
genus Anaerotruncus	WM	6	-0.090	0.187	0.91	0.008	0.910
genus Streptococcus	IVW	6	0.161	0.006	1.18	-0.071	0.366
genus Streptococcus	MR Egger	6	0.615	0.242	1.85	-0.071	0.366
genus Streptococcus	WM	6	0.110	0.120	1.12	-0.071	0.366
order Lactobacillales	IVW	6	0.175	0.000	1.19	-0.013	0.822
order Lactobacillales	MR Egger	6	0.261	0.508	1.30	-0.013	0.822
order Lactobacillales	WM	6	0.153	0.015	1.17	-0.013	0.822

Abbreviations: b represents effect size, IVW refers to inverse variance weighted, MR denotes Mendelian randomization, WM stands for Weighted median, N_IV represents the number of instrumental variables, and OR stands for odds ratio

### The relationship between gut microbiota and Influenza Pneumonia in terms of cause and effect

**Fig. 3:**
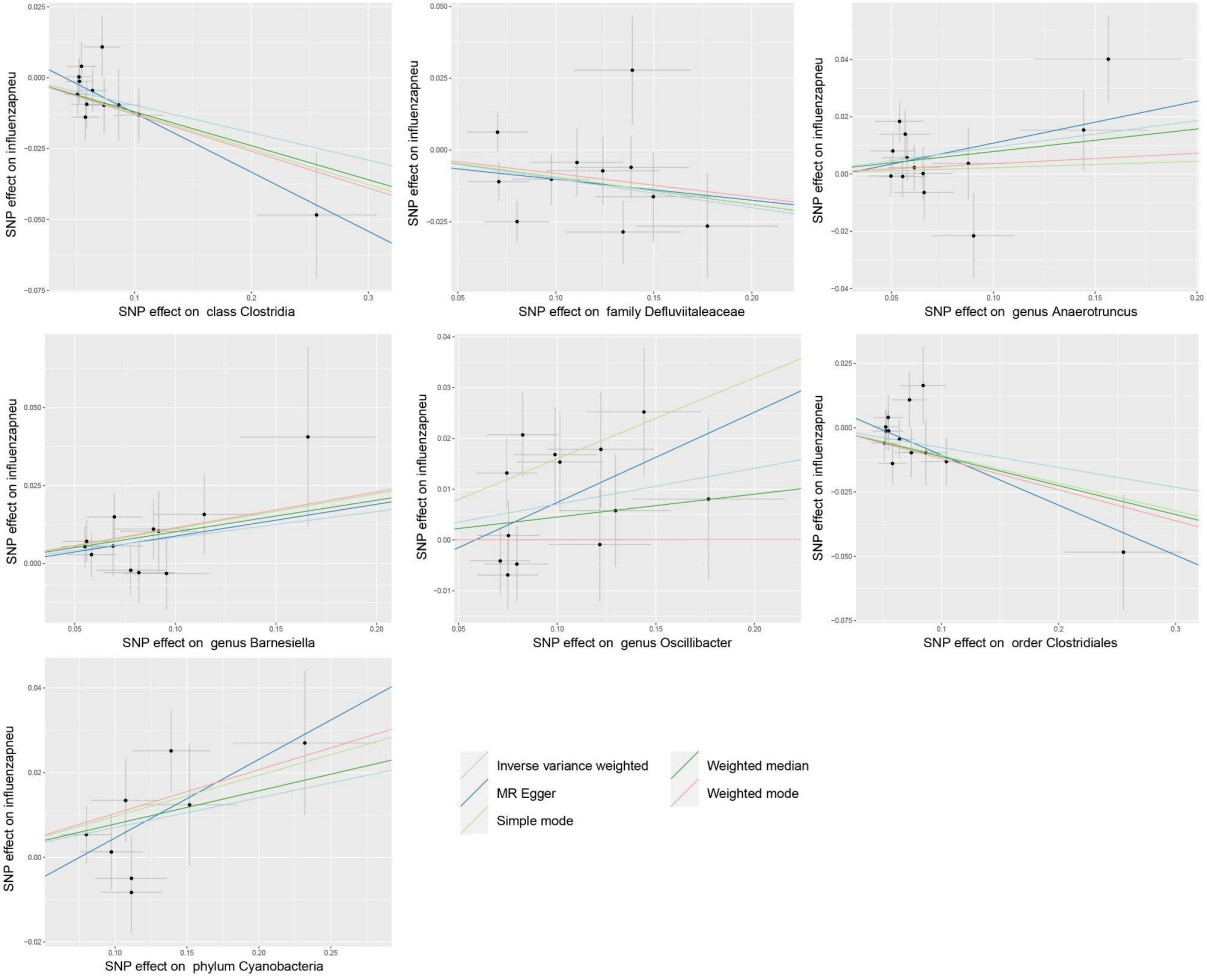
Scatter plots for the impact of gut microbiota on influenza pneumonias outcomes

Afterwards, we embarked on investigating the correlation between gut microbiome and influenza-induced pneumonia.Initially, the IVW analysis identified seven specific types of bacteria that displayed significant connections with the occurrence of influenza pneumonia. Please refer to Table 3; Fig. 3 for further details. We noticed a significant inverse relationship between the Clostridia class (OR 0.91, 95%CI 0.85 to 0.97; p-value = 0.007), Defluviitaleaceae family (OR 0.90, 95%CI 0.84 to 0.98; p-value = 0.009), and Clostridiales order (OR 0.93, 95%CI 0.86 to 0.99; p-value = 0.028) in relation to influenza pneumonias. This implies that these three bacterial categories may potentially provide defense against pneumonia. Moreover, it was discovered that the Anaerotruncus genus (OR 1.10, 95%CI 1.01 to 1.19; p-value = 0.022), Barnesiella genus (OR 1.09, 95%CI 1.01 to 1.16; p-value = 0.017), Oscillibacter genus (OR 1.07, 95%CI 1.01 to 1.14; p-value = 0.019), and Cyanobacteria phylum (OR 1.07, 95%CI 1.01 to 1.14; p-value = 0.028) exhibit a causal connection with influenza pneumonia, indicating an elevated likelihood of developing this condition.

**Table 3:** The impact of gut microbiota on influenza pneumonias outcomes

Exposure	Method	N_IV	b	p	OR	Egger_intercept	P_pleiotropy
class Clostridia	IVW	12	-0.097	0.007	0.91	0.008	0.243
class Clostridia	MR Egger	12	-0.209	0.057	0.81	0.008	0.243
class Clostridia	WM	12	-0.120	0.012	0.89	0.008	0.243
family Defluviitaleaceae	IVW	11	-0.100	0.009	0.90	-0.003	0.840
family Defluviitaleaceae	MR Egger	11	-0.073	0.609	0.93	-0.003	0.840
family Defluviitaleaceae	WM	11	-0.095	0.023	0.91	-0.003	0.840
genus Anaerotruncus	IVW	13	0.093	0.022	1.10	-0.004	0.654
genus Anaerotruncus	MR Egger	13	0.146	0.258	1.16	-0.004	0.654
genus Anaerotruncus	WM	13	0.079	0.130	1.08	-0.004	0.654
genus Barnesiella	IVW	12	0.083	0.017	1.09	-0.001	0.895
genus Barnesiella	MR Egger	12	0.102	0.492	1.11	-0.001	0.895
genus Barnesiella	WM	12	0.101	0.031	1.11	-0.001	0.895
genus Oscillibacter	IVW	13	0.071	0.019	1.07	-0.010	0.357
genus Oscillibacter	MR Egger	13	0.178	0.152	1.19	-0.010	0.357
genus Oscillibacter	WM	13	0.045	0.220	1.05	-0.010	0.357
order Clostridiales	IVW	13	-0.077	0.028	0.93	0.009	0.213
order Clostridiales	MR Egger	13	-0.195	0.066	0.82	0.009	0.213
order Clostridiales	WM	13	-0.112	0.019	0.89	0.009	0.213
phylum Cyanobacteria	IVW	8	0.070	0.028	1.07	-0.014	0.325
phylum Cyanobacteria	MR Egger	8	0.186	0.149	1.20	-0.014	0.325
phylum Cyanobacteria	WM	8	0.079	0.065	1.08	-0.014	0.325

Abbreviations: b represents effect size, IVW refers to inverse variance weighted, MR denotes Mendelian randomization, WM stands for Weighted median, N_IV represents the number of instrumental variables, and OR stands for odds ratio

The Egger intercept results demonstrated no significant directional level pleiotropy (Table 3), and the Cochran Q test did not provide evidence of heterogeneity (refer to supplementary table S4). Additionally, the single outcome analysis indicated that a single SNP had no substantial impact on the overall causal effect (refer to supplementary figure S3).

In a separate context, this analysis of MR did not offer enough proof to indicate that the quantity of intestinal microbiota is changed by influenza pneumonia (refer to supplementary Table S5).

## Discussion

This research is the initial investigation to utilize MR analysis in order to clarify the bidirectional causal connection between gut microbiota and influenza. By examining gut microbiome GWAS and metadata from large samples representing two different severity types of influenza (influenza not-pneumonia and influenza pneumonia), the study identified five bacterial taxa that have a causal impact on influenza occurrence (Table 1; Fig. 1). Additionally, seven bacterial taxa were found to have a causal effect on the development of influenza pneumonia (Table 3; Fig. 3). Moreover, a causal association was discovered between influenza infection and the abundance of seven gut bacterial taxa (Table 2; Fig. 2).

The results of this research show that the interaction between influenza and gut microbiota occurs via the gut-lung axis, which is made possible by the causal relationship between certain gut microbiota and influenza. The connection between the gut and lungs, known as the gut-lung axis, involves the influence of gut microbiota on the immune function against viral respiratory diseases [18]. Scientists have noted that soluble elements and byproducts from the gastrointestinal microbiome are capable of interacting with the respiratory system, influencing the immune reaction of the lungs towards viruses [19]. This interaction is known as the lung-gut axis [18, 19]. For instance, administering Lipopolysaccharide rectally (a molecule associated with bacteria) to mice can enhance their lung immune response and effectively enhance defense against the influenza virus [20]. Additional research on the mechanisms by which these gut microbiota impact influenza immunity will offer valuable information on utilizing the gut-lung connection for preventing and treating influenza [21].

Through the analysis of the particular bacterial categories identified in this research, it was found that the metabolism of short-chain fatty acids (SCFAs) might play a vital role in how the gut microbiota affects both influenza infection and infection-induced pneumonia [22]. Research has shown that SCFAs have a crucial impact on boosting viral immunity through the mediation of gut microbiota [23]. For instance, acetate (one of commen short-chain fatty acids) secreted by microorganisms can activate NLR Family Pyrin Domain Containing 3 (NLRP3) inflammasome via type I interferon, thereby strengthening the host’s defense against Influenza A virus [23]. This MR study identifies Actinobacteria and Clostridia as potentially protective against influenza infection. These two taxa overlap with typical intestinal probiotics involved in SCFA metabolism, such as bifidobacteria, lactobacilli, Clostridium orbiscindens, and butyricum [6, 23–25]. Notably, the MR analysis indicates that Clostridium not only exhibits potential protective effects against influenza infection but also demonstrates potential protective effects against influenza pneumonia. This implies that Clostridium has the potential to be used as a probiotic addition, not just for preventing influenza but also for inhibiting the progression of severe influenza to pneumonia.

Moreover, the analysis using MR uncovered a possible cause-and-effect relationship between flu infection and a rise in the prevalence of Anaerostipes and Lactobacillales, along with a decline in the prevalence of Anaerotruncus. The results are consistent with previous studies indicating that influenza infection could impact the quantity of gut microbiota [9, 10]. Moreover, the metabolism of SCFAs is influenced by all three of these microbiota [6, 24, 25], suggesting that changes in the abundance of certain gut microbiota due to influenza infection could potentially impact SCFA metabolism [24, 25]. Nevertheless, additional examination is necessary to comprehend the influence of influenza infection on particular gut microbiota, along with the mechanisms and consequences of SCFA metabolism.

In addition to the bacterial clusters linked to SCFA metabolism, we have also discovered additional potential connections between influenza and certain gut microbiota. Initially, we discovered a cause-and-effect relationship between contracting the flu and a rise in the prevalence of Bacilli class. The findings align with others’ results [24, 26, 27]. For instance, Zhang et al. [26] on Bacillus subtilis, in which an increased abundance of endogenous B. animalis in the gut of mice enhanced their resistance to influenza. Therefore, the immune effect of class Bacilli on influenza, particularly in severe cases, merits further investigation. Additionally, Gierse et al. [27].observed a significant increase in Staphylococcaceae within class Bacilli in H1N1 influenza-infected mice compared to normal and COVID-19 infected mice. This implies that the plentiful presence of Bacilli may potentially function as indicators in the intestines for the identification of influenza and other infections.

Secondly, MR analysis demonstrated a bidirectional causal relationship between Streptococcus and influenza, which has been supported by other studies. For instance, Tsang et al. [28]. found that a tenfold increase in Streptococcus abundance resulted in a 48% and 63% decrease in host susceptibility to influenza A and B, respectively, in a study on influenza transmission within families. Multiple epidemiological studies [29–31] have also indicated that an increase in Streptococcus abundance following influenza infection can lead to secondary streptococcal pneumonia. These findings align with our results.

Furthermore, the analysis using MR revealed a potential link between influenza and influenza pneumonia with the presence of specific gut bacterial groups, including Romboutsia, Tyzzerella3, Defluviitaleaceae, and Barnesiella. It is worth noting that these bacterial groups have not been extensively studied in the context of respiratory infectious diseases. Further research is necessary to investigate the influence of these bacterial groups on influenza, as highlighted by these findings. This research will aid in our comprehension of the connection between gut microbiota and influenza, as well as the formulation of approaches for preventing and treating influenza.

This study still has some limitations. Initially, certain scientists hold the view that influenza pneumonia has the potential to alter the abundance of intestinal microbiota [31]. However, the incongruous OR value in this investigation renders the cause-and-effect connection between influenza pneumonia and the alteration of Gut microbiota abundance impracticable (Supplementary table S5). This impracticability could potentially be attributed to the inadequate sample size of Gut microbiota subtypes. Secondly, the MR analysis results obtained from the GWAS cohort, which primarily consisted of individuals with European ancestry, may have relevance to the European population. Further validation is needed to extend this result to other ethnic groups. Furthermore, while the results obtained from IVW were confirmed by the sensitivity analysis using the median approach of MR, the validation outcomes of MR egger did not align with IVW. Hence, additional verification is required to confirm the cause-and-effect connection established in this research.

To summarize, the MR analysis carried out in this research uncovers a possible two-way causal connection between the prevalence of particular gut bacteria and influenza infection. Furthermore, this study showed substantial proof backing the causal association between the abundance of certain gut microbiota and the development of pneumonia caused by influenza. Further exploration of the mechanisms that govern the interaction between particular bacterial communities and influenza can greatly aid in the efforts to prevent, monitor, and treat influenza.

### Electronic supplementary material

Below is the link to the electronic supplementary material.

Supplementary Material 1

Supplementary Material 2

Supplementary Material 3

Supplementary Material 4

Supplementary Material 5

Supplementary Material 6

Supplementary Material 7

Supplementary Material 8

## Data Availability

The datasets generated during analysis in the current study are available in the the International MiBioGen Alliance [13], [https://mibiogen.gcc.rug.nl/], accessed 2023 May 7th and the FinnGen consortium [14], [https://r8.finngen.fi/ ],accessed 2023 May 9th .

## List of abbreviations

MR: Mendelian randomization
GWAS: genome-wide association study
mbQTL: microbiome trait loci
mbBTL: mapping binary trait loci
IVs: Independent variables
SNPs: Single-nucleotide polymorphisms
LD: Linkage Disequilibrium
MAF: minor allele frequency
IVW: inverse variance weighting
WM: weighted median
OR: odds ratio
b: effect size
CI: confidence interval
N_IV: the number of instrumental variables
SCFAs: short-chain fatty acids
NLRP3: NLR Family Pyrin Domain Containing 3
