# Supplementary material for: Association between gut microbiota and influenza: a bidirectional two-sample mendelian randomization study
Source: BMC Infect Dis. 2023 Oct 17;23:692. doi: 10.1186/s12879-023-08706-x (PMC10580584; doi:10.1186/s12879-023-08706-x)
Supplement: Supplementary file 2 — Supplementary Material 2 [file 12879_2023_8706_MOESM2_ESM.pdf]

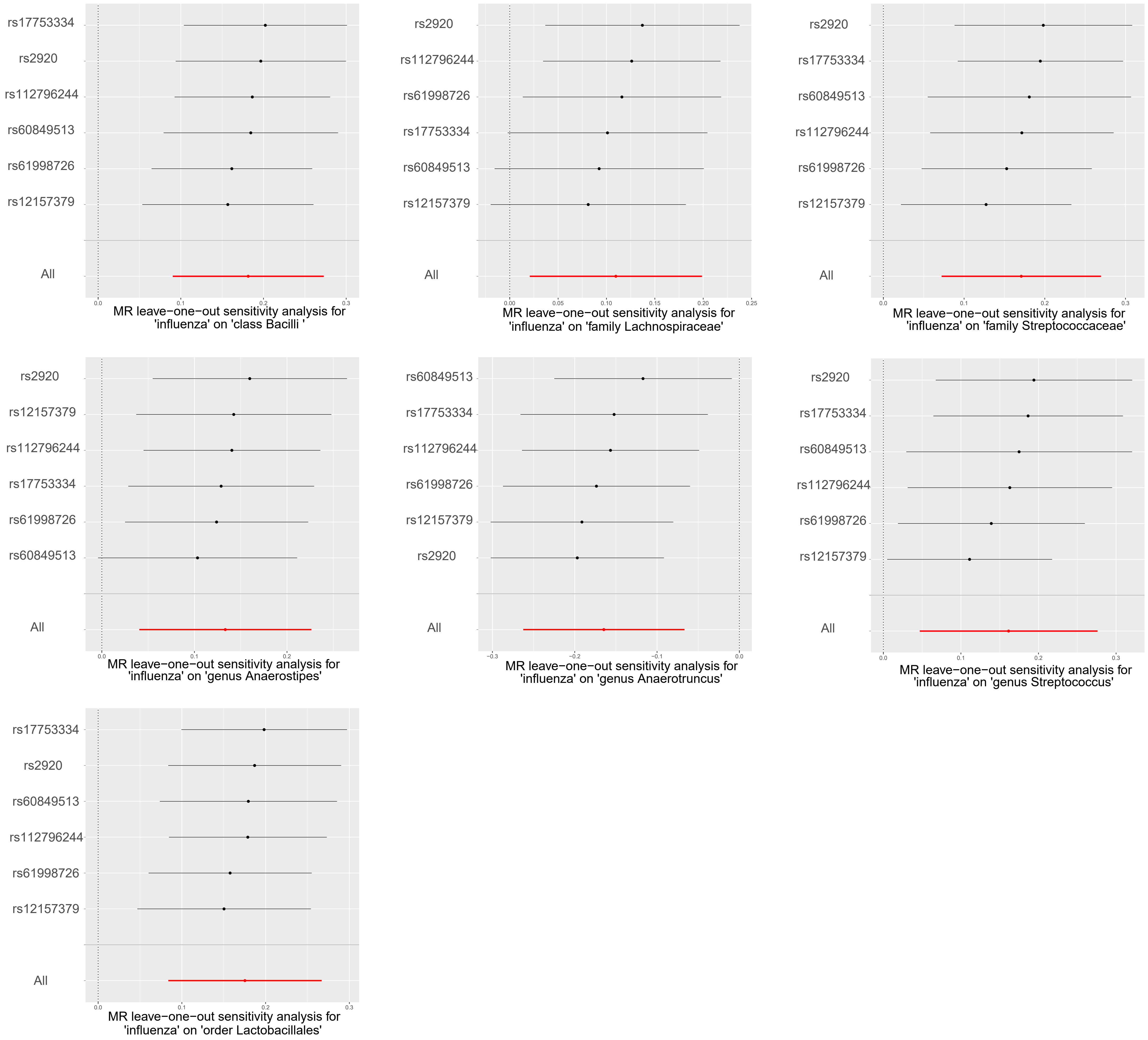

supplementary figure S2: Leave-one-out plots for the causal association between influenza (not-pneumonias) and gut microbiota
