# Supplementary material for: Association between gut microbiota and influenza: a bidirectional two-sample mendelian randomization study
Source: BMC Infect Dis. 2023 Oct 17;23:692. doi: 10.1186/s12879-023-08706-x (PMC10580584; doi:10.1186/s12879-023-08706-x)
Supplement: Supplementary file 3 — Supplementary Material 3 [file 12879_2023_8706_MOESM3_ESM.pdf]

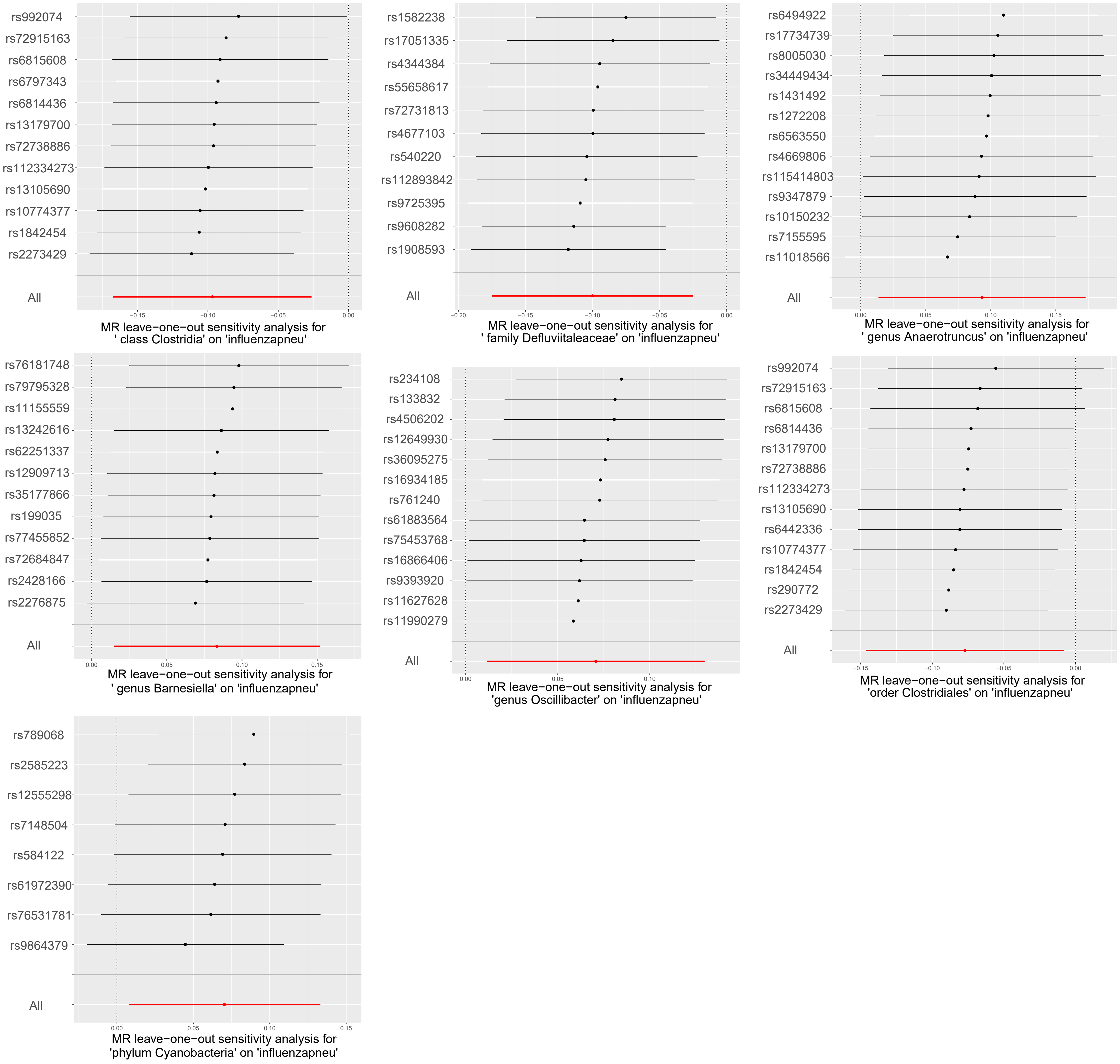

supplementary figure S3: Leave-one-out plots for the causal association between gut microbiota and influenza pneumonias
